# Penile Angiokeratomas (PEAKERs): An exceedingly Rare Clinical Variant

**DOI:** 10.5826/dpc.1104a121

**Published:** 2021-10-01

**Authors:** Jaime Piquero-Casals, Daniel Morgado-Carrasco, Juan Francisco Mir-Bonafé, Eduardo Rozas-Muñoz

**Affiliations:** 1 Department of Dermatology, Dermik Multidisciplinary Dermatological Clinic, Barcelona, Spain; 2 Department of Dermatology, Barcelona Hospital Clínic, Barcelona University, Spain; 3 Department of Dermatology, Son Llàtzer Hospital, Palma de Mallorca, España; 4 Department of Dermatology, San Pablo Hospital de, Coquimbo, Chile

## Case Presentation

**Figure 1:**
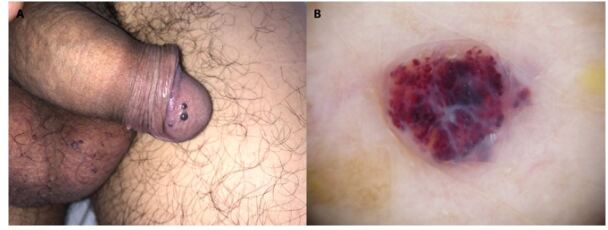
(A) Multiple 1–3 mm purple papule on glans and scrotum of 5 years’ duration. (B) Dermoscopy of angiokeratoma revealing multiple dark blue and red lacunae, together with erythema.

An otherwise healthy 29-year-old man presented with multiple 1 to 3 mm purple papules present in the last 5 years on his glans and scrotum (Figure 1A). He had no history of sexually transmitted diseases, nor he experienced trauma to the area. Dermoscopy revealed dark blue and red lacunae, together with erythema. Dermoscopic analysis confirmed angiokeratoma diagnosis (Figure 1B). After explaining the benign nature of the lesions, the patient refused to receive any treatment and the papules remained stable in number at a 6-month follow-up visit.

## Teaching Point

Genital angiokeratomas are benign vascular lesions occurring most commonly on the scrotum or vulva.

The differential diagnosis of angiokeratomas includes common nevus, blue nevus, primary melanoma, or cutaneous metastasis, angiomas, as well as others lesions with vascular patterns, such as basal cell carcinomas, and pyogenic granulomas. When angiokeratomas are diffusely present, evaluation for a lysosomal storage disease (LSDs) (for instance, Fabry disease) and possible referral to a clinical geneticist should be considered [1].

Penile angiokeratomas (PEAKERs) are an uncommon subtype of genital angiokeratomas [2]. These benign vascular tumors typically presenting as multiple lesions on the corona glans penis. Treatment includes cryotherapy and lasers, among others. Dermoscopy should be included as a part of the clinical inspection to avoid unnecessary invasive investigation [2].
